# Longitudinal myelin MR imaging in patients with multiple sclerosis: a narrative review

**DOI:** 10.1007/s00415-026-13655-x

**Published:** 2026-02-11

**Authors:** Lis J. M. van den Boogaard, Agata Bochman, Gerhard S. Drenthen, Stephanie A. M. Knippenberg, Simone Monachino, Oliver H. H. Gerlach, Jacobus F. A. Jansen

**Affiliations:** 1 Department of Radiology and Nuclear Medicine, Maastricht University Medical Center+, Maastricht, the Netherlands; 2 Mental Health and Neuroscience Research Institute, Maastricht University, Maastricht, the Netherlands; 3 Department of Neurology, Academic MS Center Zuyd, Zuyderland Medical Center, Sittard-Geleen, the Netherlands; 4 Department of Electrical Engineering, Eindhoven University of Technology, Eindhoven, the Netherlands; 5 Department of Research, Development and Education, Epilepsy Center Kempenhaeghe, Heeze, the Netherlands

**Keywords:** Multiple sclerosis, MRI, Myelin, Longitudinal

## Abstract

Progression of multiple sclerosis (MS) remains incompletely understood. MS is characterized by demyelination, resulting in a wide variety of symptoms. Conventional magnetic resonance imaging (MRI) is the current standard for diagnosing and monitoring patients. However, conventional MRI has its limitations in visualizing the myelin dynamics. In contrast, advanced myelin-specific MRI techniques enable non-invasive, in vivo quantification of myelin content. Such approaches hold promises for the early detection of pathology and for improving the understanding of disease mechanisms and their relationship to clinical outcomes, particularly since many patients with MS experience progression of symptoms that cannot be fully explained by conventional imaging measures. This narrative literature review aims to summarize recent advances in longitudinal myelin-specific MRI studies in MS and their clinical applications. Overall, longitudinal studies demonstrated that myelin-specific MRI techniques can capture dynamic changes in myelin, possibly aiding in understanding the progression of MS, although inconsistencies persist both between and within techniques. Even though more myelin-sensitive than myelin-specific methods, such as diffusion MRI or multi-contrast methods, are not specific to demyelination, they could aid clinical follow-up by predicting lesion formation, as changes are visualized before being present on conventional MRI. These findings underscore the need for future research that integrates MRI-derived metrics with detailed assessment of disease courses.

## Introduction

Multiple sclerosis (MS) is a chronic disease characterized by inflammation and demyelination within the central nervous system (CNS). This pathological process leads to damage of the myelin sheath, an insulating layer surrounding axons essential for efficient conduction of electrical impulses [1]. Demyelination is a hallmark in MS, and since it can occur in various areas of the CNS, it contributes to the heterogeneity of clinical manifestation [2]. MS predominantly manifests in two forms: relapsing–remitting multiple sclerosis (RRMS) and progressive multiple sclerosis (PMS). Patients with RRMS typically experience periods of symptom worsening, known as relapses, followed by partial or complete recovery, while patients with PMS worsen without those relapses [3]. The remitting period may be attributed to a combination of resolution of inflammation, neuroplasticity, and remyelination processes [4], which thus appears significantly impaired in PMS [5]. Nonetheless, many patients with RRMS exhibit gradual neurological deterioration between relapses, a phenomenon referred to as progression independent of relapse activity (PIRA) [6]. Understanding these disease progression types is crucial for prevention strategies and slowing down the disease.

Current diagnostic protocols rely heavily on clinical assessments and the identification of white matter lesions using conventional anatomical magnetic resonance imaging (MRI) [7]. In patients with RRMS, relapses are frequently associated with new white matter lesions caused by inflammation, which can be visualized on MRI with gadolinium-based contrast agents [8]. In contrast, in PMS, the formation of new lesions is less common [8]. Instead, PMS is characterized by the presence of slowly expanding or chronic active lesions, the growth of which is more difficult to visualize due to their gradual expansion.

The progression of neurological impairment in MS is commonly evaluated using standardized clinical scales. The most widely applied measure is the Expanded Disability Status Scale (EDSS) score [9], which is considered the golden standard for assessing the disability status in MS. Other tools for assessing the progression of patients with MS are the No Evidence of Disease Activity-3 score (NEDA-3) status, the Paced Auditory Serial Addition Test (PASAT-3) [10], and the Multiple Sclerosis Functional Composite (MSFC), comprising the Symbol Digit Modalities Test (SDMT), the Timed 25-foot Walk (T25FW) [11], and the 9-Hole Peg Test (9-HPT).

The progression of both RRMS and PMS symptoms correlates only modestly with conventional MRI findings [3]. Although conventional MRI is extremely sensitive to white matter lesions, it lacks specificity for assessing myelin content. Its sensitivity to diffuse or subtle brain changes is limited, and it cannot differentiate between inflammatory demyelination and remyelination processes [12]. Consequently, MRI findings often correlate only partially with patients’ functional outcomes and levels of disability [13, 14]. Additionally, conventional MRI cannot measure subtle changes in myelin content, which could be an important contributor to the clinical progression of the disease. Therefore, imaging techniques that enable direct visualization and quantification of myelin loss and repair hold exciting potential for improving early lesion detection and monitoring disease progression.

Advances in imaging technology have introduced new opportunities to overcome these limitations of conventional MRI, particularly through the development of non-invasive, in vivo myelin-specific MRI techniques. Each technique employs a distinct mechanism to quantitatively evaluate myelin, potentially offering new insights into the relationship between demyelination and disease progression [15, 16]. Given the chronic course of MS and the importance of monitoring progression, applying these advanced imaging methods in longitudinal studies could enhance the understanding of MS pathology and support clinical decision-making.

This narrative literature review summarizes recent findings in longitudinal myelin imaging studies in MS and their clinical relevance. First, an overview of the different myelin-specific imaging methods used across the included articles is provided. Second, findings from longitudinal studies are described, emphasizing their ability to capture disease progression and relate imaging measures to clinical outcome, an era often underrepresented in existing cross-sectional reviews. Finally, a perspective for future research of longitudinal myelin changes in MS is provided.

## Methods

This narrative literature review outlines prior work on longitudinal MRI studies in patients with MS focusing on the myelin content. PubMed was searched for articles with the following terms in either the title or abstract: “multiple sclerosis” or “MS”, “longitudinal”, “magnetic resonance imaging” or “MRI”, and “myelin” or “myelin imaging”. The PubMed search was conducted until October 2025, which resulted in 110 articles being published between 2005 and 2025. These publications were screened using the title, followed by their abstract. Non-English articles and literature reviews were excluded. Furthermore, only human studies with longitudinal MRI measurements and a follow-up period of at least 1 year were considered for this review. Additionally, the selected articles were cross-referenced to include as many relevant studies as possible. The articles included were fully read.

## Myelin-specific imaging methods

Four well-established quantitative MRI approaches for assessing myelin are: (i) myelin water imaging, (ii) magnetization transfer imaging, (iii) quantitative susceptibility mapping, and (iv) diffusion-weighted imaging techniques [16]. Furthermore, additional methods identified in this review include multi-contrast techniques which combine multiple conventional scans, enhancing their sensitivity to myelin content. Understanding these methods is critical for interpreting the longitudinal changes in MS pathology. An overview of the methods mentioned is shown in Table 1.

**Table 1:** Overview of the mentioned myelin-specific MRI methods

Category		Technique		Measure	Abbreviation	What it measures	Demyelination	Remyelination
Myelin water imaging		Myelin water imaging		Myelin water fraction	MWI/MWF	Proportion of water trapped between the myelin bilayers	↓	↑
Magnetization transfer imaging		Magnetization transfer imaging		Magnetization transfer ratio	MTI/MTR	Exchange of energy between free water and bound protons	↓	↑
				MT saturation	MTsat	Corrected exchange of energy between free water and bound protons	↓	↑
				Myelin volume fraction	MVF	Proportion of myelin volume in voxel	↓	↑
Quantitative susceptibility mapping		Quantitative susceptibility mapping			QSM	Change of susceptibility	↑	↓
		χ-separation		Diamagnetic maps		Change of susceptibility of myelin	↑	↓
Diffusion-weighted imaging		Diffusion tensor imaging		Radial diffusivity	DTI / RD	Diffusion perpendicular to the axonal fibers	↑	↓
				Fractional anisotropy	FA	Directionality (anisotropy) of diffusion	↓	↑
		Q-space myelin mapping			qMM	Diffusion restriction around axons	↓	↑
Multi-contrast methods		T_1w_/T_2w_-ratio				T_1_ and T_2_ relaxation	↓	↑
		Combined myelin estimation			CME	T_1_ and T_2_^*^ relaxation times	↓	↑

Changes of the values of the mentioned method are shown in the de- and remyelination columns, where an increase (↑) or decrease (↓) indicates how this measure changes regarding de- and remyelination

### Myelin water imaging

**Fig. 1:**
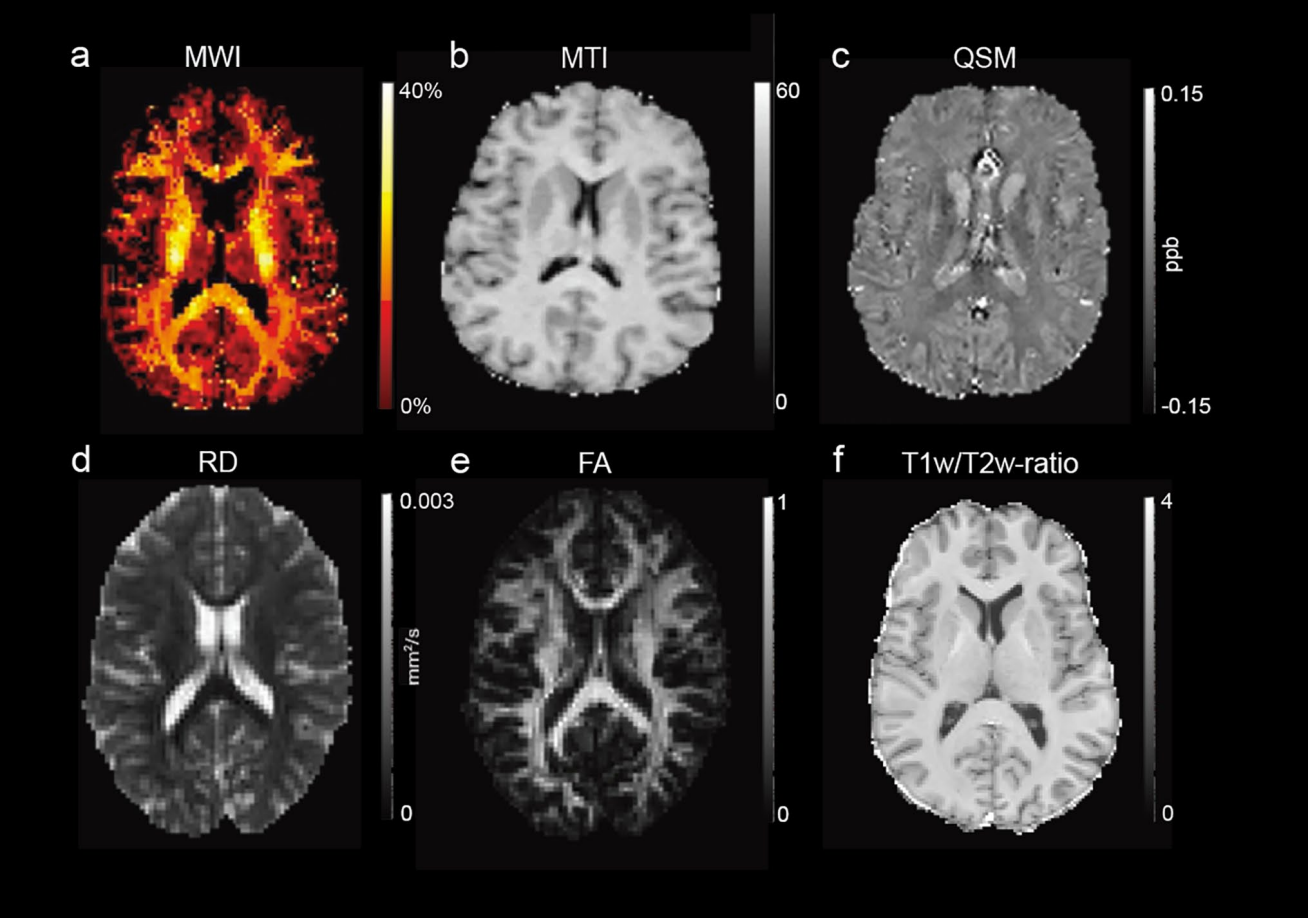
Examples of the myelin-specific MRI methods discussed in this review. **a** Myelin water fraction (MWF) map, **b** magnetization transfer ratio (MTR) map as shown in [37], **c** quantitative susceptibility mapping (QSM) reconstruction, **d** diffusion tensor imaging (DTI) radial diffusivity (RD), **e** fractional anisotropy (FA) map, and f) T_1w_/T_2w_-ratio. A and C-F are constructed from data of the authors’ research group. B is adapted under compliance with the CC-BY 4.0 license

Due to the layered structure of myelin, water is trapped between the bilayers. Myelin water imaging (MWI; Fig. 1A) can isolate the MRI signal of this trapped water based on its relaxation properties. Water molecules in myelin water are more restricted, compared to intra- and extracellular water [17], wherefore their relaxation times are shorter. By measuring the characteristic T_2_, T_2_^*^, or T_1_ relaxation times of the different water components, the myelin water component of a certain voxel in the brain can be quantified [18]. Subsequently, the quantified myelin content is calculated as the myelin water fraction (MWF), which is the myelin water component as a fraction of the total water signal [17–19]. Even though the MWF does not quantify the myelin sheath directly, the myelin water component is specific to myelin changes and has been validated using histology in prior studies [20]. Compared to normal regional MWF values, a lower MWF signifies demyelination, while a higher MWF reflects preserved or restored myelin.

### Magnetization transfer imaging

Magnetization transfer imaging (MTI; Fig. 1B) assesses magnetization exchange between free water protons and a macromolecular proton pool, to which myelin is a major contributor. In conventional MRI, the protons bound to macromolecules are invisible due to their short T_2_ relaxation time. However, by applying additional radio frequency (RF) pulses, MTI saturates bound protons, transferring their magnetization to free protons and reducing tissue signal [21, 22]. The magnetization transfer ratio (MTR) quantifies this effect by comparing the images taken before and after applying the RF pulses [22]. More advanced approaches, such as MT saturation (MTsat) and myelin volume fraction (MVF) mapping, further improve specificity by accounting for relaxation effects and improving sensitivity to macromolecular-related contrast while reducing confounding relaxation effects [23, 24]. Low MTI values reflect reduced capacities of magnetization exchanges, indicating damage to the myelin, whereas high values are an indication of normal levels of myelin content.

### Quantitative susceptibility mapping

Quantitative susceptibility mapping (QSM; Fig. 1C) quantifies magnetic susceptibility-weighted images (SWI) by reconstructing spatial susceptibility distributions from phase data acquired with gradient-echo MRI [25, 26]. QSM is particularly effective in visualizing iron deposition and other secondary changes associated with demyelination. In MS this is shown, for instance, by visualizing high-contrast rims around active chronic demyelinating lesions [25]. Therefore, QSM is not myelin specific, but rather shows secondary effects of myelin changes. Higher contrast in QSM indicates more deviation from standard magnetic susceptibility. In terms of MS, increased susceptibility may reflect iron accumulation and/or loss of diamagnetic tissue components, such as myelin, which can be a secondary cause of demyelination.

Χ-separation was introduced to be able to differentiate between iron and myelin. In χ-separation, the individual contribution of the paramagnetic susceptibility sources, such as iron, is separated from the diamagnetic susceptibility sources, such as myelin [27]. Especially the diamagnetic susceptibility source maps are of interest for this review, which is what we refer to when discussing χ-separation, unless stated otherwise. The diamagnetic maps are on a negative scale, where a lower, negative value indicates more myelin.

### Diffusion-weighted imaging

Diffusion-weighted imaging (DWI) techniques, including diffusion tensor imaging (DTI), measure the random motion of water molecules within tissue. The mobility of water molecules can be restricted in certain directions due to tissue microstructure (anisotropic) or move freely in all directions (isotropic) [28]. Measuring the diffusion of water in several different directions allows to characterize the tissue microstructure using tensor analysis. This approach results in the subdivision of axial diffusivity (AD), which represents the diffusivity parallel to the principal diffusion direction, and radial diffusivity (RD: Fig. 1D), which represents the diffusivity perpendicular to the principal diffusion direction [29]. One of the most commonly used metrics in diffusion imaging is the fractional anisotropy (FA; Fig. 1E), representing the level of anisotropic diffusion within a voxel between zero (completely isotropic) and one (completely anisotropic) [28]. Potential causes of increased isotropic diffusion of tissue are edema, inflammation, or demyelination. Diffusion-weighted measures are sensitive to restrictions in the movement of the water molecules, and therefore relate to general microstructure and are not specific to only changes in the myelin content. RD or FA values are often used as indirect markers for the myelin content, where increases in the RD and decreases in the FA would relate to demyelination, although the RD and FA are influenced by multiple other microstructural factors.

Q-space myelin mapping (qMM) can be used as a derivative of diffusion-weighted imaging. By quantifying the multilevel, multi-shell diffusion, a percentage of myelin change can be calculated [30]. Compared to other diffusion indices and T_2_-weighted scans, qMM showed increased contrast in depicting myelinated tracts [30]. Additionally, this technique allow distinction between remyelinating lesions and chronic lesions. A loss of qMM signal was indicated as demyelination, where an increase of myelin signal indicates remyelination.

### Combination of multiple scans into one modality

Multi-contrast methods enhance the myelin sensitivity of conventional scans. For example, the T_1w_/T_2w_-ratio (Fig. 1F) has been introduced as a myelin proxy [31]. This ratio divides the intensity values of T_1_-weighted (T_1w_) images by those of T_2_-weighted (T_2w_) images, which are both generally readily available in clinical cohorts. T_1w_- and T_2w_-images are generally evaluated separately. However, when combined, they can provide a better estimate of relative myelin contrast maps. In these maps, the ratio enhances the sensitivity to the myelin signal, as the intensity of myelin is bright on the T_1w_-images, and dark on the T_2w_-images [31]. Research has shown that the T_1w_/T_2w_-ratio is as sensitive to the myelin content as MTR and more sensitive than DTI [32–34]. While myelin sensitivity is increased in the T_1w_/T_2w_-ratio, myelin specificity is not, and thus rather represents a more general measure of tissue microstructure [35]. A more myelin-specific multi-contrast method is the combined myelin estimation (CME) maps. In CME, T_1_ and T_2_^*^ relaxation time maps are combined through a spatial independent component analysis [36]. This analysis extracts the myelin-specific signal of both maps to increase myelin specificity compared to both maps separately. In both multi-contrast methods, higher signal intensities indicate higher values of myelin, whereas lower signal intensities reflect myelin loss.

## Search results

After completing the search and implementing the exclusion criteria, 39 articles were included in this review (Table 2). The MRI scanners used in the selected studies had a field strength of 3 Tesla unless noted otherwise (*/**). The selected articles are classified into four subcategories: (i) myelin changes in non-lesional tissue in MS, (ii) myelin changes in lesional tissue in MS, (iii) myelin imaging in relation to use of medication, and (iv) clinical predictors for myelin changes in MS. The results are discussed per disease subtype (RRMS and PMS).

**Table 2:** Clinical information on selected studies

Article information			MS patients’ demographics						HC demographics			Procedures	
Cited number	Author	Year	Sample size	Sex	Mean age at T0 (y)	Disease subtype	Mean EDSS at T0	Mean disease duration (y)	Sample size	Sex	Mean age at T0 (y)	Times measured MS	Myelin-specific MRI method
[39]	Boaventura et al	2022	22	14F, 8 M	36.5	RRMS	1.5	8.9				T0, 1y	T_1w_/T_2w_
[40]	York et al	2022	79	48F, 14 M	37.6	RRMS	2.0	1.3	12	7F, 5 M	44.0	T1, 1y	MTR, MTsat
[41]	Klistorner et al	2018	43	24F, 19 M	42.1	RRMS	1.4	5	20	12F, 8 M	41.0	T0, 3.5y	DTI
[42]	Cooper et al	2021	102	68F, 34 M	33.2	Early MS/CIS	1.5	0.35	50	34F, 16 M	31.8	T0, 2-6y	T_1w_/T_2w_
[43]	Levesque et al	2010	5	5F	42.6	RRMS	-	-	5	2F, 3 M		Monthly until 5 m, 8, 11 m	Quantitative MTI, MWI
[44]	Vandeleene et al	2023	17	7F, 10 M	36.0	11 RRMS, 6 PMS	2.5	3.4				T0, 2.5y	MTsat
[45]	Barletta et al	2021	25	21F, 4 M	38.2	RRMS	2	3.2	19	10F, 9 M	35.8	T0, 1y	CME**
[46]	Vavasour et al	2018	11	7F, 4 M	38.2	RRMS	0.5–6	8.5	4	3F, 1 M	42.8	T0, 4.8y	MWI
[47]	Fox et al	2011	21	15F, 6 M	41.6	18 RRMS, 3 SPMS	-	11.9				T0, 1y	DTI
[48]	Harrison et al	2011	78	52F, 26 M	43.6	40 RRMS, 24 SPMS, 14 PPMS	3.5	10.8				T0, 3mRRMS, 6 m, yearly 1-4y	DTI, MTR, qT_2_
[49]	Andersen et al	2018	31	22F, 9 M	77	13 SPMS, 11 RRMS, 7 CIS	6.5	50	5	3F, 2 M	55.0	T0, 1-60y	DTI
[50]	Hayton et al	2012	117	146F,54 M	50.5	SPMS	6.0	20.1				T0, 1, 2y	MTR*
[52]	Xie et al	2025	33	23F, 10 M	34.3	RRMS	2.5	3.7	34	21F, 13 M	31.4	T0, 1.45y	χ-separation
[53]	Hagemeier et al	2018	120	81F, 39 M	44.2	98 RRMS, 22 SPMS	2.5	12.8	40	24F, 16 M	43.7	T0, 2y	QSM
[52]	Cagol et al	2024	127	81F, 46 M	45.6	81 RRMS, 26 SPMS, 20 PPMS	2.5	6.1	73	39F, 34 M	36.0	T0, 2y	MVF, QSM
[54]	Lazzarotto et al	2024	140	96F, 44 M	38.2	37 CIS, 71 RRMS, 32 PMS	2.0	5.4	84	55F, 29 M	34.3	T0, 1y	MTR
[55]	Huerta et al	2024	15	12F, 3 M	42.4	RRMS	3	8.5				T0, 0.5y, 1y	MWI**, qMT**
[56]	Brown et al	2014	19	17F, 2 M	17.1	RRMS	-	4				T0, 3, 6, 12 m, yearly OR T0, 1y, 2-4y	MTR*
[57]	Vargas et al	2015	23	16F, 7 M	32.8	20 RRMS, 3 CIS	1.5	5.3	11	-	-	T0, 2-12 m	MWI
[58]	Kitzler et al	2022	12	8F, 4 M	33.2	Included 4 weeks after first relapse	1.6	max 4 weeks	4 / 60	3F, 1 M / 39F, 21 M	35.6 / 39.7	T0, 3 m, 6 m, 1y	MWI*
[59]	Shin et al	2025	23	16F, 7 M	33.0	RRMS	1.0	1.75				T0, yearly – 3.3y	χ-separation
[60]	Vavasour et al	2009	2	2F	43.0	RRMS	2.75	9.5				T0, 2, 4, 6, 12 m	MWI*
[61]	Zhang et al	2016	29	-	36.5	RRMS	-	-				T0, 0.1-2y	QSM
[62]	Ontaneda et al	2014	21	15F, 6 M	41.6	RRMS	-	11.9				T0, 1, 2, 6, 12, 18 m	DTI
[63]	Muller et al	2024	168	101F, 67 M	47.0	98 RRMS, 46 SPMS, 24 PPMS	3.0	6.2	103	57F, 46 M	33.0	T0, 2y	χ-separation
[64]	Chawla et al	2018	9	7F, 2 M	54.1	4 RRMS, 1 PPMS, 4 SPMS	3.3	15.7				T0, 2.4y	QSM**
[65]	Pandya et al	2020	48	29F, 19 M	42.3	39 RRMS, 9 SPMS	2.5	0.8				-6 m, T0, 2y	MWI
[66]	Bonnier et al	2017	23	15F, 8 M	35.7	RRMS	1.6	2.8	9	4F, 5 M	34.2	T0, 2y	MTR
[67]	Tonietto et al	2023	19 / 40	13F, 6 M / 22F, 18 M	32.0 / 49.0	19 RRMS / 12RRMS, 18 PMS	2 / 4.5	7.4 / 8.4	8 / 39	5F, 3 M / 23F, 16 M	32.0 / 41.0	T0, 2-4 m, 1y	MTR
[68]	Rahmanzadeh et al	2022	49	27F, 13 M	43.0	32 RRMS, 8 PMS	2.5	8.9	76	45F, 31 M	35.0	-3 m, T0, 2y	QSM
[69]	Calvi et al	2022	135	99F, 36 M	35.5	RRMS	1.5	5.5				T0, 2.9, 6.5y	MTR
[70]	Vavasour et al	2025	50	31F, 19 M	38.0	RRMS	2.0	4.0	23	-	-	T0, 24, 48, 96, 144, 192w	MWI, MTR
[71]	Tagge et al	2022	25	19F, 6 M	57.0	18 RRMS, 6 SPMS, 1 PPMS	2, 4.5, 6.5	-				-6 m, T0, 6 m, 1y	DWI, MTsat
[73]	Preziosa et al	2021	55	33F, 22 M	37.0	RRMS	2.0	10				T0, 6 m, 1y, 2y	MTR, T_1w_/T_2w_
[74]	Kitagawa et al	2024	48	37F, 11 M	35.2	RRMS	1.5	5.9				T0, 6 m, 1y	qMM
[75]	Tanikawa et al	2017	24	13F, 11 M	37.3	RRMS	2.3	-				T0, 3, 6, 9 m, 1y, 1.25y	qMM
[76]	Kolind et al	2022	55	34F, 22 M	38.0	RRMS	2.0	3.5	23	15F, 8 M	37.0	T0, 24, 48, 96, 144, 192w	MWI
[77]	Vavasour et al	2019	42	28F, 14 M	35.0	RRMS	3.0	6.1				T0, 0.5, 1, 1.5, 2y	DTI
[78]	Dimov et al	2022	12	-	40.0	-	-	7.6				T0, 1y	QSM, MWI

*F female; M male; MS multiple sclerosis; RRMS relapsing–remitting MS PMS: progressive MS; PPMS: primary PMS; SPMS secondary PMS; CIS clinically isolated syndrome; EDSS: Expanded Disability Status Scale; HC: healthy controls; y: years; m: months; w: weeks; DTI: diffusion tensor imaging; DWI: diffusion-weighted imaging; CME: combined myelin estimation; MTI: magnetization transfer imaging; MTR: magnetization transfer ratio; MTsat: MT saturation; MVF: myelin volume fraction; MWI: myelin water imaging; QSM: quantitative susceptibility mapping; SWI: susceptibility weighted Imaging; T: tesla; T0: baseline.*

*: 1.5 T

**: 7 T

## Myelin changes in non-lesional tissue in MS

### Normal-appearing white matter (NAWM) changes

On conventional anatomical MRI scans, white matter regions appearing unaffected by disease-related activity or demyelination are termed normal-appearing white matter (NAWM) [38]. Nevertheless, advanced MRI techniques can reveal subtle changes within these areas.

Depending on the MRI method used, varying results of (de)myelination over time were reported in patients with RRMS. Patients with RRMS showed stable myelin content levels in NAWM over 12 months, according to the T_1w_/T_2w_-ratio [39] and MTR [40]. Furthermore, a longer follow-up of 3–4 years showed a stable AD, RD, and FA in patients with RRMS [41], and an even longer follow-up of 6 years showed stable T_1w_/T_2w_-ratio values in early MS patients [42]. However, comparing the myelin content in NAWM between healthy volunteers and patients with RRMS did show differences. Specifically, at baseline, myelin content in NAWM of the patients with MS was significantly lower compared to healthy controls in the same age range, according to a more advanced quantitative MTI [43], MTsat [44], CME [45], and MWF [43, 46]. While NAWM of healthy volunteers showed no significant changes after a follow-up of at least 5 years, patients with RRMS exhibited a steady annual decrease in myelin content in NAWM, according to FA values in DTI [47, 48], MTR values [48], including MTsat [40], and MWI with decreasing myelin content of up to -17% [46]. Notably, most studies did not consider individual disease courses. When accounted for, patients with minimal symptoms exhibited increasing MTsat values over time, consistent with remyelination [44]. Overall, the different MRI methods show conflicting results, where myelin-specific and less myelin-specific methods show either stable or decreasing myelin levels in NAWM. These discrepancies might be caused by other mechanisms, disease duration, follow-up duration, or the lack of considering the disease course of the patients.

Patients with PMS showed comparable results as the patients with RRMS. Lower myelin content was found compared to healthy volunteers in the same age range according to MTsat [44], FA [47–49], and RD values in DTI [49]. This lower myelin content, measured with FA, further decreased over follow-up in patients with PMS, while it remained stable in the healthy controls [47, 48]. Notably, two studies included both patients with RRMS and PMS and did not differentiate between these subtypes in the results [44, 47], which could have biased the results. However, Harrison et al. [48] did make this distinction and did not find any significant differences in baseline and follow-up DTI indices, but they did find a faster decline in the myelin content of patients with PMS using MTR. Looking specifically at the body of the corpus callosum, differences between patients with RRMS and PMS were found. The values of the RRMS patients were in the range of the healthy controls, where the values of the PMS patients indicated more demyelination according to FA and RD DTI-indices [49]. This discrepancy between results could indicate a location-specific difference between disease subtypes, which is not observable in the whole NAWM.

Clinical measures provide further insight into changes in NAWM in patients with MS. In clinically stable or improving patients with RRMS or PMS, indicated by the NEDA-3 scores, the myelin content of NAWM appeared to increase over time. This increase was measured with the T_1w_/T_2w_-ratio [42] and MTsat [44] and suggested potential remyelination. Increasing EDSS correlated to decreasing FA and increasing RD indices in the corpus callosum [49]. Specifically looking at patients with RRMS, the baseline T_1w_/T_2w_-ratio was negatively correlated with the baseline EDSS scores [39]. Contrarily, changes in MWF values were not correlated with changes in EDSS scores [46]. Zooming in on patients with PMS, longitudinal, positive correlations have been reported between the MTR and the MSFC, PASAT-3, and 9-HPT, but not the EDSS score [50]. These positive correlations indicate that improvements in clinical symptoms are associated with increased myelin content.

### Gray matter (GM) changes

Overall, myelin content is lower in gray matter (GM) than in white matter reflecting the lower density of myelinated axons in GM. Given that demyelination is a hallmark of MS, myelin-related alterations are typically less pronounced in GM than in white matter. In GM, normal appearing tissue is not as easy to establish as in white matter; specific scan protocols are needed to make sure that there are no lesions present in the cortical and deep GM. Since none of the cited studies have used these, the term GM is used instead of normal appearing GM.

Longitudinal studies in RRMS show conflicting results about myelin levels in GM over time. Patients with RRMS using disease-modifying treatment showed a stable T_1w_/T_2w_-ratio in GM over a 12-month follow-up [39]. However, in clinically stable or improving patients, an increase in myelin content was observed in cortical GM, but not in deep GM, as measured by MTsat [44]. This increase could be attributed to the repair mechanisms of patients responding to the treatments. Comparing patients with healthy controls showed a lower myelin content, according to χ-separation, in healthy controls versus patients with RRMS at baseline in the caudate, putamen, and hippocampus [51]. However, no longitudinal changes were found in all deep GM structures. In contrast, deep GM regions, such as the caudate and putamen, showed increasing magnetic susceptibility over time [52]. These QSM changes indicate potential pathological alterations beyond demyelination happening in deep GM, such as iron deposition which is common in those areas.

Further longitudinal myelin changes have been observed in deep GM structures of patients with PMS. A recent study reported changes in the MVF in the thalamus in patients with active PMS [53]. These patients exhibited a faster decline in myelin content in the thalamus compared to healthy volunteers, inactive PMS, and RRMS, where patients with a higher overall T_2_ lesion volume at baseline had an even more accelerated thalamic MVF reduction [53]. Additionally, in a small group of patients with PMS, a tendency towards increased magnetic susceptibility in the caudate was measured with QSM over a 2-year follow-up [52]. While patients receiving disease-modifying treatments exhibit increased myelin content in cortical GM, no such changes are detected in deep GM regions, according to MTsat [44]. This distinction raises the question whether treatment effects are limited to specific areas or whether methodological limitations mask deeper pathological changes. Untreated patients with PMS, for example, show no significant changes in GM myelin content measured using MTR [50]. This may reflect either disease stability or insensitivity of certain MRI techniques to detect subtle variations.

The relationship between myelin alterations in GM and clinical disability remains unclear, as studies report conflicting findings. Barletta et al. [45] found no correlations between the EDSS score and CME in cortical GM of patients with RRMS. Similarly, Hagemeier et al. [52] did not find a correlation using QSM in deep GM. Even though Cagol et al. [53] found similar results at baseline; they did find a correlation between the EDSS progression and decreased magnetic susceptibility, measured with QSM in deep GM. A positive correlation between the EDSS and the myelin maps of χ-separation was found in the putamen, hippocampus and amygdala, at baseline and at follow-up, however, this was not longitudinally tested [51]. According to MTI, greater cortical demyelination at baseline, a PMS phenotype, and the number of relapses increased the risk of clinical progression after 5 years [54]. A greater extent of cortical remyelination decreased this risk, but only in patients with a shorter disease duration. The correlation between the SDMT and deep GM changes in patients with RRMS varied between hemispheres. In the right hemisphere, the superior and inferior parietal, lateral occipital, and postcentral cortex were positively correlated with the SDMT [45]. In the left hemisphere, the rostral middle frontal, superior frontal, superior parietal, and posterior cingulate cortex showed this positive correlation. However, Barletta et al. [45] is the only study investigating the relationship between myelin measures and the SDMT and has a relatively small sample size (n = 25).

## Myelin changes in lesional tissue in MS

Lesions can be categorized into classical active lesions, chronic inactive lesions, chronic active or slowly expanding lesions (SELs), and black hole lesions (BHLs). Active lesions are characterized by inflammation and blood–brain barrier disruption [8]. Inflammation activity, such as relapses and formation of new lesions, decreases in PMS, but neurodegeneration and progressive decline increase. These active lesions are typically highlighted on contrast-enhanced T_1_-weighted scans and are more prevalent in RRMS. As inflammation subsides, most lesions do not fully remyelinate and transition into chronic inactive lesions. Lesions that maintain low-level inflammatory activity over time are classified as chronic active lesions (CALs) or SELs, characterized by a slowly expanding core that requires long-term follow-up to detect [8]. BHLs, distinguishable by their hypo-intensity on T_1_-weighted scans, reflect severe axonal loss post-demyelination [55]. Differentiating these lesion types may provide insights into distinct disease progression trajectories.

### Classical active lesion changes

Newly formed active lesions are highlighted on contrast-enhancing T_1_-weighted scans. In patients with RRMS, gadolinium-enhanced lesions exhibit lower myelin content compared to NAWM on MTI- [43, 56], and MWI-scans [43, 57, 58], and in the T_1w_/T_2w_-ratio [39]. Additionally, newly visible lesions on the diamagnetic χ-separation map showed this demyelination in most lesions [59]. This reduced myelin content is accompanied by an increased total water content, as evidenced by changes in MWF [60]. Over time, an increase in MWF and MTI indicates partial remyelination, although values rarely return to pre-lesion levels [43, 56, 57]. The lower MWF values in gadolinium-enhanced lesions compared to non-enhanced lesions suggest the effects of acute inflammation on demyelination. Furthermore, a QSM study [61] showed that the majority of enhancing lesions transition to non-enhancing states or disappear entirely over a two-year follow-up, reflecting blood–brain barrier repair and less inflammation. During the transition from enhancing to non-enhancing lesion, there was an increase in magnetic susceptibility. However, persistent hyper-intensity in these lesions may indicate secondary processes, such as iron accumulation, which QSM cannot directly differentiate from myelin changes. Χ-separation can make this distinction. Only looking at the diamagnetic map showed that, indeed, most lesions (50%) remyelinated, 23% remained stable and 35% further demyelinated [59]. However, this study did not investigate the longitudinal iron changes, wherefore no conclusive statements can be made about the iron and myelin processes after lesion formation.

**Fig. 2:**
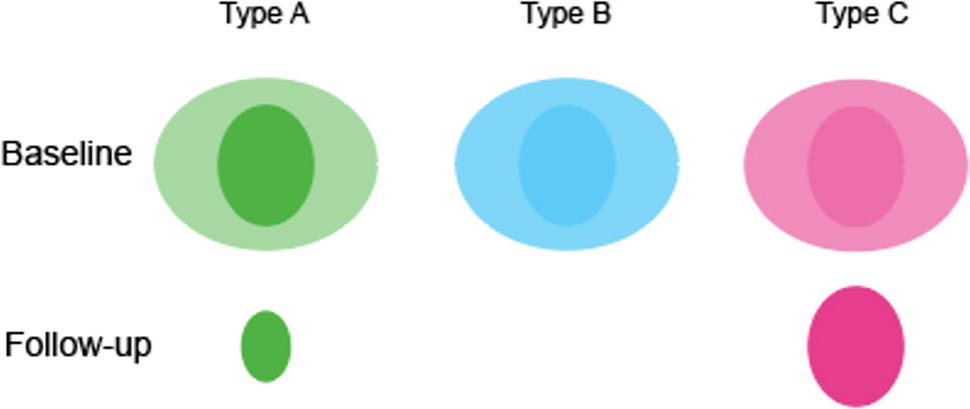
Representation of the three lesion types and their courses over time as mentioned in [60]. The color intensity indicates the amount of demyelination, with high demyelination indicated as more intense

Dividing lesions into core and periphery regions can aid understanding of the growth trajectories. Vavasour et al. [60] combined MWF and water content measures to characterize these trajectories in patients with RRMS and classified lesions into three types: A, B, and C (Fig. 2). Type A lesions are characterized by a “black hole” core and decreased peripheral MWF. Over follow-up, the total lesion diminished in size but did not fully disappear, MWF increased, and the periphery returned to normal. This pattern suggests an inflammatory and demyelinating process, followed by remyelination. Type B lesions demonstrated transient MWF reduction in both core and periphery, which normalized after follow-up, indicating inflammation with associated edema. Type C lesions exhibited persistent MWF reduction in the core, with a recovered periphery after follow-up, indicative of sustained demyelination. Larger lesions were more likely to exhibit the heterogeneity seen in types A and C, whereas smaller lesions showed more homogeneous MWF changes [58, 60]. These findings strengthen the statement that MWF is a reliable measure to make precise distinctions in terms of myelin content trajectories.

Peripheral regions of lesions may also serve as early indicators of myelin changes in patients with RRMS, as these regions are at risk and might already display subtle changes. MTsat measures decrease months prior to lesions' visibility on conventional MRI, while NAWM remains stable [44]. According to the T_1w_/T_2w_-ratio, there was already a decreased myelin content visible before the lesions became visible on conventional MRI scans [39]. Similarly, DTI measures subtle change months before conventional imaging. Specifically, FA decreases approximately 3 months before gadolinium enhancement on T_1w_-scans [62]. The decrease in FA before lesion enhancement may reflect early microstructural changes, such as axonal damage or initial demyelination, preceding visible lesion development on conventional MRI.

Lesion evolution trajectories are linked to patient-specific clinical outcomes. Improvements in cognitive and motor functions in patients with PMS, measured by EDSS, MSFC, PASAT-3, and 1/9HPT, are predicted by increases in MTR, suggesting remyelination [50]. Contrarily, higher EDSS scores at baseline were associated with future remyelination according to χ-separation [59]. Alternatively, demyelinating lesions, as measured by MWF, are associated with disability worsening, with lesion type C specifically associated with relapse occurrences and poorer cognitive outcomes [60]. The number of studies investigating the correlations between clinical measures and myelination patterns is low, but the available results do show a noteworthy consistency.

### Chronic inactive lesion changes

A considerable proportion of CNS lesions in MS are chronic and remain visible on anatomical scans for years. Myelin-specific MRI techniques provide insights beyond conventional imaging, revealing subtle changes within these lesions, not detectable on conventional MRI.

Evidence from myelin-specific MRI techniques remains inconclusive concerning the long-term stability versus progressive degeneration of myelin within chronic inactive lesions. A susceptibility study shows no significant myelin changes in healthy controls over time [63]. In the majority of lesions of patients with MS, changes were neither found in patients with RRMS nor PMS over a follow-up of 2 years, suggesting that most lesions stay inactive after they have been formed [63, 64]. This stability was independent of disease activity and worsening of symptoms in the patients. Contrarily, only looking at patients with RRMS and longitudinal tracking of chronic lesions with DTI revealed progressively decreased FA values over 3–4 years [41]. These results were strengthened by the use of more myelin-specific MRI metrics, such as MTsat [40], MWF [65], and MTR [40, 66], which found an overall decrease of myelin over a two-year follow-up. Additionally, higher MTR values at baseline were correlated with a higher chance of lesion resolution after a follow-up of 2 years [66]. However, the change of MTR was not significant when only patients with RRMS were studied [40]. The progress of lesions may differ between RRMS and PMS disease courses. However, this distinction was not addressed in all included studies, therefore no definitive conclusions can be drawn.

Analyzing individual lesions within a person, rather than through group averages, provides deeper insights into lesion evolution. Notably, most lesions (43–75%) remained stable, 13–41% of lesions displayed significant demyelinating trajectories, and 7–16% exhibited remyelinating patterns, according to CME [45], MWF [65], and MTR [66]. It was shown that patients with PMS had more demyelinating lesions than patients with RRMS [65]. Additionally, subdividing the lesions into core and perilesional areas showed significant differences compared to NAWM in MTsat values [44]. Demyelination was largest in the core, where the perilesional areas’ progression was smaller and the myelin in NAWM remained stable [41, 44]. This variability highlights the heterogeneity of lesion development as the lesions appeared similar at baseline.

Dividing lesions based on either gray or white matter lesions revealed different myelination patterns. Remyelinating patterns were mostly found in white matter lesions according to MTR in patients with RRMS [66]. Also, χ-separation analyses showed these patterns by a decrease in susceptibility in white matter lesions over time, with a larger change in patients with RRMS, suggesting more extensive remyelination compared to PMS [63]. In contrast, cortical lesions demonstrated stability over time according to χ-separation [63]. Examining cortical lesions with MWF showed mostly demyelinating patterns with spontaneous remyelination in almost half of the lesions [54]. The demyelinating cortical lesions predicted long-term clinical progression, which was prevented by cortical remyelination. At baseline, CME revealed lower values in cortical lesions of patients with RRMS compared to contralateral GM and corresponding regions in healthy controls but could not distinguish between the demyelinating or remyelinating patterns [45].

Additionally, lesion location with regard to the ventricles appeared to influence myelination levels. Chronic lesions located closer to the ventricles showing greater myelin loss according to MWI [54, 65], and MTI [67]. Higher remyelination was found at a larger distance from the ventricles. However, existing periventricular remyelination was associated with lower EDSS at baseline and a lower change in EDSS over a 5-year follow-up [67]. Also, χ-separation made this association between remyelinating lesions and lower EDSS change [63]. Additionally, more paramagnetic rim lesions were found around the ventricles according to QSM [68]. The observed correlation between proximity to the ventricles and increased myelin loss may indicate a gradient of vulnerability within the CNS, possibly correlated to the cerebrospinal fluid.

### Slowly expanding lesion (SEL) changes

SELs, a subtype of chronic lesions, represent an early stage in the progression to chronic and black hole lesions. MTI studies show that SELs have lower myelin content than non-SELs, with progressive myelin loss over time [55, 69, 70], and was confirmed showing comparable results using MWF [70]. This decline correlates with the clinical disability in RRMS, where higher EDSS scores are associated with increased SEL counts, larger SEL volumes, greater inflammation, neurodegeneration, and more BHLs [69]. Since 96% of the SELs become BHLs [55], they serve as markers of disease severity and contribute to worsening of symptoms in RRMS, as is measured with the EDSS, even without relapses [69]. Developing earlier disability, measured as confirmed disability progression (CDP), increased the risk fivefold for every one-unit increase of SEL volume [69]. Additionally, patients with PMS may have more SELs, potentially explaining their higher symptom burden. Therefore, assessing SELs could help guide treatment strategies.

### Black hole lesion (BHL) changes

BHLs are associated with severe demyelination and axonal damage. A previous study found that 43% of contrast-enhancing lesions at baseline progressed to BHLs after one year [47]. Higher RD values predicted the conversion to a BHL at follow-up [47]. Additionally, BHLs are strongly linked to SELs, as 96% of the SELs were also classified as BHLs, and SELs were more frequently found within BHLs than in non-BHLs [55]. BHLs show complete myelin loss, evidenced by marked signal reduction in MWI. While partial remyelination is possible, full recovery is rare. Studying the areas of lesion development retrospectively showed lower MVF in lesions which transitioned into BHLs compared to lesions that did not [71]. Additionally, the decrease in MVF after lesion development was larger in BHLs compared to non-BHLs indicating more long-term myelin loss [71]. Although direct studies linking BHL load to clinical outcomes are lacking, the strong overlap with SELs allows for inferences regarding their impact on disease progression. Disease-modifying treatments (DMTs) may reduce disability and enhance the potential for remyelination.

## Myelin imaging in relation to use of medication

There are various DMTs for the treatment of MS, which primarily aim to decrease the severity and number of relapses and prevent further brain lesion formation [72]. These can be subdivided into low-, moderate-, and high-efficacy DMTs.

### Low- and moderate-efficacy medication

Moderate- and low-efficacy medications, such as fingolimod, Interferon B1a (IFNβ-1a), dimethyl fumarate (DMF), and glatiramer acetate (GA), have been investigated with regard to longitudinal myelin changes in patients with MS. Fingolimod is an immunomodulator reducing lymphocyte counts in the blood. It is shown that in patients using fingolimod the T_1w_/T_2w_-ratio and MTR increase over 2 years, especially in NAWM [73]. In GM, initial decline was followed by recovery, indicating long term tissue repair. In a qMM study [74], remyelination was detected in 6 of 14 fingolimod treated patients. Additionally, patient response varied, with optimal responders (42%), improved EDSS scores or remaining disability-free, showing remyelination without new damage [75]. Suboptimal responders (58%) exhibited concurrent demyelination, reinforcing the statement that baseline disease severity influences repair outcomes. Contrarily, the patients with remyelinating patterns were not the same patients having a NEDA score [75] or EDSS improvements [74]. This suggests that remyelination is not necessarily accompanied by clinical improvements and that therapeutic effects may rely more on immunomodulation than direct myelin repair.

IFNb-1a was evaluated in SELs over a 4-year period with MTR and MWF [76]. IFNb-1a showed greater MWF decline than ocrelizumab, both in NAWM and the chronic lesions. This highlights IFNb-1a’s limited protective capacity in terms of myelin and MWF’s sensitivity to progressive damage. If patients subsequently switched from IFNb-1a to ocrelizumab, results improved, further supporting the IFNb-1a’s weaker remyelination potential [76]. Patients treated with DMF showed similar changes for remyelination (45.5%) as patients treated with fingolimod [74]. GA, which is the only low-efficacy DMT in this review, has only shown EDSS improvement and remyelination in one of four participants [74]. These findings emphasize how subtle myelin differences detected by MWF, MTR, and qMM do not necessarily align with clinical outcomes.

### High-efficacy medication

High-efficacy medication, such as natalizumab, alemtuzumab, and ocrelizumab, have been studied using advanced imaging techniques assessing their effects on myelin integrity in patients with RRMS. Natalizumab, leading to reduction of lymphocyte migration into the CNS, showed early increases of the myelin content as measured with the T_1w_/T_2w_-ratio and MTR within the first 6 months of treatment for white matter lesions [73]. It was followed by a gradual decrease and plateau, suggesting the primary action of natalizumab being instant damage stabilization rather than active remyelination. Both T_1w_/T_2w_-ratio and MTR in NAWM and GM remained stable throughout the entire course of treatment, however, if also the patients remained stable is unknown [73]. Nevertheless, in a qMM study comparing natalizumab with moderate-, such as fingolimod, DMF, and low-efficacy medication, such as GA, natalizumab has shown the highest remyelination rate (62.5%) [74]. However, qMM-detected remyelination did not correlate with EDSS improvements as only 12.5% showed EDSS improvement. Contrarily to MTR and MWF, qMM can provide a more detailed view of de- and remyelination, and thus the lesion dynamics.

The effect of alemtuzumab and ocrelizumab, both DMTs utilizing monoclonal antibodies, on myelin was assessed with MWF. Alemtuzumab stabilized MWF in lesions and NAWM over 24 months [77]. When ocrelizumab was compared with a lower-efficacy medication, IFNb-1a, smaller MWF declines were shown in SELs of the ocrelizumab group [70, 76]. Additionally, the MWF in NAWM stabilized or increased after DMT initiation or switching from IFNb-1a to ocrelizumab. These findings support ocrelizumab’s role in lesion expansion reduction and preventing demyelination. Although MWF increases in NAWM, recovery in chronic lesions remained limited [76].

## Demographic predictors for myelin changes in MS

Currently, the disease course of MS is unpredictable. It is therefore important to continue the search for potential key factors influencing the disease course. The longitudinal MRI studies included in this review have found myelin-related biomarkers and characteristics related to the course of MS. Those studies also found demographic differences that influenced myelination during the MS disease course.

The most reported characteristic related to the course of MS was age, without consensus between the different myelin-specific MRI methods. According to MWI [57], MTI [67], and qMM [74], baseline age was not related to the amount of myelin. Contrarily, younger patients with MS are more likely to experience remyelinating trajectories, according to DTI [49], MTI [56], χ-separation [63], and qMM [74, 75]. Specifically, MS onset during childhood or adolescence often results in no physical disability in the first 10 years of the disease, despite having a comparable lesion burden to adults [56]. This resilience may be attributed to the faster remyelination and greater neural plasticity in younger individuals. The discrepancy between the different studies about the influence of age on the amount of myelin might be attributed to differences in the mean and range of age of the cohorts, as myelination in healthy brains shows a clear age effect.

Secondly, a vital role of sex was identified, which is important as more females are diagnosed with MS than males. According to a study employing qMM, women had a higher chance of remyelination as the majority of the remyelination group consisted of female participants (86.4–100%), while all male participants using DMF were categorized into the non-remyelination group [74]. Additionally, χ-separation found this benefit of the female sex in chance of future remyelination [59]. However, χ-separation [63] and MTI [67] could not make this distinction. The DTI [41] and qMM [74] studies suggest a more severe disease course for males.

Finally, the effect of disease duration was assessed regarding the rate of demyelination over follow-up. No correlations have been found that suggest an influence of disease duration according to QSM [52], χ-separation [63], qMM [74], and MTI [67]. Another MTI-study did not find the association between spontaneous remyelination and disease course, but they did find that patients with a longer disease course had a lower myelin content and more demyelination over follow-up [54]. Only the T_1w_/T_2w_-ratio was negatively correlated to disease course [39]. An explanation for the lack of association between disease duration and rate of re- and demyelination is the heterogeneous disease course and the inclusion of mainly patients with RRMS.

## Discussion

The aim of this review was to provide a detailed overview of longitudinal changes in myelin content in MS, as measured by various myelin-specific MRI techniques. In NAWM, patients with MS, particularly progressive MS, consistently show a decline in myelin content over time, as detected by myelin-specific techniques. Less specific methods, such as the T_1w_/T_2w_-ratio, often fail to detect such changes. Associations between general clinical scores and myelin-specific imaging findings are inconsistent, as hypothesized. Although more functionality specific scales, such as the MSFC, show better alignment with imaging markers, these are understudied. In GM, myelin changes are more subtle compared to NAWM. Therefore, findings vary between increased cortical myelin in stable or improving patients, while deep GM regions show increases in magnetic susceptibility, suggesting iron accumulation rather than remyelination. Clinical correlations in GM remain unclear due to the inherently low myelin content and different imaging sensitivities. For lesion analysis, active lesions initially show strong demyelination, followed by partial remyelination over time, especially in RRMS, most notably captured using MWI and MTI. Chronic inactive lesions show heterogeneous patterns, where some remain stable, some continue to demyelinate, and others remyelinate, where MWI or MTI are preferred for distinguishing these features due to their high myelin specificity. Lesion location appears relevant, with periventricular lesions showing greater myelin loss, which also explains the greater demyelination found in the corpus callosum. SELs, which almost always evolve into BHLs, are associated with increased clinical disability. Demographic factors such as age, sex, and disease duration show mixed results, with younger patients and women potentially having more favorable remyelination patterns.

### NAWM changes

Findings on NAWM changes in MS demonstrate both the potential and limitations of current MRI techniques in detecting subtle disease-related alterations. Although advanced imaging methods consistently demonstrate reduced myelin content in patients with MS compared to healthy controls, the clinical significance and consistency of these changes remain uncertain. Myelin-specific measures, such as MWF and MTR, consistently reveal progressive loss, whereas less myelin-specific methods, such as the T_1w_/T_2w_-ratio, often fail to capture such changes. DTI, though sensitive, remains incapable of detecting myelin changes directly. Changes in FA may equally reflect axonal injury or reorganization rather than demyelination itself, complicating interpretation. Furthermore, the inability of several studies to clearly distinguish between RRMS and PMS populations further complicates interpretation and potentially confounds the results [44, 47]. Progressive patients are more likely to exhibit continuous NAWM decline, wherefore mixing subtypes may mask disease-specific patterns. Additionally, the weak correlations observed between myelin-specific MRI markers and EDSS underscore a broader limitation of the EDSS, namely its insensitivity to subtle functional decline. In contrast, stronger associations between alternative clinical scores (MSFC, PASAT-3, and 9-HPT) and MTR and MTsat suggest that more scores may better capture minor changes in myelin content linked to functional performance in MS. Taken together, these findings emphasize that advancing the understanding of NAWM pathology in MS requires not only enhanced imaging specificity, but also alignment with clinical measures that can capture the functional impact of subtle demyelination.

### GM changes

Interpretation of GM changes is challenging due to its inherently low myelin content, which limits measurement sensitivity and increases variability across studies. The T_1w_/T_2w_-ratio lacks specificity, yet its agreement with MTR supports the view that cortical myelin is stable. In contrast, MTsat shows inconsistent cortical increases, raising the question of whether this reflects true remyelination or methodological sensitivity. QSM adds further complexity because its signal results from the combined effect of not only myelin but also iron and calcium. Increases in susceptibility within deep GM are therefore more likely to indicate iron accumulation as secondary effects of demyelination rather than remyelination. This statement was confirmed by the introduction of χ-separation, which indeed did not find any longitudinal myelin changes in deep GM structures. Therefore, not all observed changes truly reflect myelin alterations but rather other pathological processes in MS. Clinically, these inconsistencies limit the reliability of myelin changes in GM as biomarkers. Understanding these discrepancies requires careful consideration of the strengths and limitations of each MRI method and the biological complexity of GM changes in MS.

### Lesional changes

Myelin-specific approaches, such as MWI and MTI, provide the clearest characterization of classical active lesions, enabling precise characterization of lesion trajectories, particularly in RRMS. Even though the lack of specificity in DTI and T_1w_/T_2w_-ratio, it serves as an effective predictor for lesion emergence on conventional MRI. However, this reduces their utility or understanding lesion biology. Also, QSM and CME lack myelin specificity in distinguishing between inflammation and demyelination. Dimov et al. [78] tried to resolve this issue using a Gradient Echo (GRE) sequence to distinguish iron accumulation from demyelination in a single voxel. Results showed a noteworthy correlation with the QSM-iron and MWF maps, which would make detection of demyelination easier [78]. Also, χ-separation, a newly emerging technique, shows the promising capability to make distinction. The predominance of RRMS-focused studies in the literature may skew insights, even though this skewness would be expected since active lesions are less prevalent in PMS.

Findings of chronic inactive lesions highlight how different MRI sequences provide complementary, yet sometimes conflicting insights into their pathology. While FA suggests demyelination, DTI’s lack of myelin specificity means observed changes could stem from axonal degeneration. QSM, despite showing no longitudinal myelin changes, correlates well with MWF, where higher susceptibility aligns with lower myelin content [68]. Explanations for the different results are that iron accumulation remains stable after lesion formation, where diffusion metrics decrease because of other mechanisms such as axonal breakdown. More myelin-specific techniques, such as MWF and MTR, provided refined insights, identifying distinct lesion trajectories, depending on lesion location and baseline characteristics. These findings emphasize the heterogeneity of chronic lesions and their potentially important location regarding the cerebrospinal fluid.

Currently, clinical associations with chronic lesion trajectories remain limited, as only one χ-separation study investigated and found a correlation between remyelinating lesions and a reduced EDSS in RRMS and PMS [63]. However, current studies on chronic inactive lesions have not investigated the differences between PMS and RRMS, which may also be a key factor influencing the heterogeneity in the patterns of these lesions. Additionally, lesion location and the number of SELs and BHLs may influence the disease severity. However, studies investigating this in terms of myelin-specific MRI methods are scarce, due to the long follow-up time of SELs.

### Medication usage

Studies investigating treatment effects indicate that different MRI methods vary in their ability to capture myelination patterns. For natalizumab, qMM appears most sensitive, detecting early repair that was not consistently observed with MWF or MTR. This may reflect the higher specificity of qMM for myelin integrity, but also the strong anti-inflammatory profile of natalizumab, which facilitates repair. In contrast, fingolimod and other moderate-efficacy therapies show weaker remyelination signals, which may result from limited biological effect rather than methodological insensitivity.

MWF has been effective in tracking myelination during treatment with alemtuzumab and ocrelizumab, particularly in aggressive phases of the disease. The T_1w_/T_2w_-ratio indicated possible structural recovery with fingolimod and has shown sensitivity to the onset of repair, which complements qMM’s specificity for confirming remyelination regions. Taken together, these results highlight that treatment-related imaging changes cannot be attributed solely to technique performance, but also reflect the mechanisms of action and efficacy of the therapies studied. However, systematic comparisons across therapies and imaging techniques remain limited.

### Methodological considerations

Methodological variability and limitations across the reviewed literature pose substantial challenges to interpreting the dynamics of myelin changes in MS. A commonly used framework divides patients into RRMS and PMS, yet it remains debatable whether this division sufficiently reflects the underlying biological processes [79], particularly regarding (re)myelination. Additionally, many studies do not stratify results by subtype or include both without clear justification, potentially obscuring key differences in disease progression and response to therapy [44, 47]. Similarly, the classification of lesions into chronic active versus chronic inactive forms lacks standardization. While the concept of SELs offers promise for understanding smoldering inflammation, their detection and tracking require prolonged follow-up, which is lacking in most studies. As a result, these lesion types are often underrepresented or misclassified, and conclusions about their clinical impact remain speculative. Another way for understanding smoldering inflammation is by looking at perilesional areas, separating these areas from the NAWM.

Additionally, the variability in imaging methodology significantly affects study comparability. Techniques range from highly myelin-specific methods, such as qMM, MWI and MTI, to more indirect or less specific metrics, such as DTI, QSM, or T_1w_/T_2w_-ratio. While each method has merits, inconsistencies within and between methods in application, field strength, post-processing, and myelin specificity compromise cross-study interpretation in terms of MS. Even within the same myelin-specific MRI method, it is recognized that scanner and protocol differences can result in different outcomes [80]. This limitation can account for the disagreements between studies. In longitudinal studies, this becomes especially as every time point must be scanned on the same scanner with the same software version to be sufficiently comparable. Regardless of the increased correlation to clinical variables, these considerations, together with the many technically demanding preprocessing steps, complicate the translation to the clinical care of patients with MS. After proving to be useful for the clinical care of patients with MS, these techniques should be translated to the clinic. However, the support of the MR vendors is necessary to facilitate this process.

Inconsistencies also comprise cross-study interpretation in the comparison of the different treatments in terms of myelin, and few studies have investigated this. Besides, associations between myelin changes and clinically relevant changes, such as stability or worsening of patients’ symptoms, are lacking, complicating the interpretation. Clinical correlations are also inconsistently reported, and when included, often rely heavily on the EDSS, which may not capture refined changes in cognitive or motor function. Alternative measures, such as 9-HPT or PASAT-3, are used less frequently and used in small subgroups. However, using those more specific tests examining one specific aspect of the symptoms may give additional information compared to a composed score, such as the EDSS.

Furthermore, many studies involve small cohorts and short follow-up periods, often limited to 1 or 2 years. These are insufficient to capture the slow trajectories of demyelination, remyelination, or lesion evolution, particularly in chronic lesions and SELs. Without long-term data, distinguishing between temporary fluctuations, which are common in RRMS, and true pathological progression remains difficult. In addition, demographic variables such as age, sex, and disease duration are rarely analyzed in depth, despite preliminary findings suggesting they may influence remyelination potential.

### Recommendations for future research

Taken together, the methodological limitations in current longitudinal myelin imaging studies in MS highlight the need for more long-term research efforts. One important future direction involves reassessing the conventional subdivision between RRMS and PMS and instead considering a spectrum-based approach that incorporates radiological, immunological, and remyelination profiles. Similarly, lesion classification could be improved through consensus criteria for chronic active, chronic inactive, and SELs, supported by validated imaging biomarkers. These refined classifications should be integrated with harmonized imaging protocols, particularly those that combine myelin-specific techniques, such as MWI and MTI, with structural MRI and advanced post-processing of conventional MRI. Additionally, more myelin-specific methods shall emerge in longitudinal MS studies. For example, more advanced diffusion models, such as neurite orientation dispersion and density imaging (NODDI) [81], can provide additional information about the pathology of MS. Future studies should ensure sufficiently long follow-up periods, ideally 5 years or more, to capture gradual demyelination and remyelination, especially in SELs, periventricular lesions, and perilesional areas. Furthermore, larger, and more diverse patient cohorts, including untreated individuals and those with later-stage PMS, are essential to improve generalizability.

Another key direction lies in the development of clinically translatable, sensitive MRI markers of progression. Multi-contrast approaches show potential for detecting subtle changes in myelin content using standard clinical MRI sequences, before visibility on one conventional MRI scan. For patients, shorter scanning times are preferred. Therefore, developing sufficient post-processing methods of conventional MRI would be extremely beneficial to visualize and possibly predict disease worsening. These methods could be further validated against more specific techniques, such as MWI, in longitudinal designs. A promising study would combine quantitative MRI with conventional MRI, integrating multiple cognitive and motoric tests with serum biomarkers. Lastly, the impact of demographic factors (such as age, sex, hormonal status) and individual treatment responses on remyelination potential should be explored further. Such comprehensive and longitudinal studies are critical to move beyond descriptive imaging toward predictive and personalized monitoring of MS progression.

## Conclusion

Longitudinal studies demonstrate that myelin-specific MRI techniques can capture myelin dynamics in MS, whereas less specific measures yield inconsistent results, limiting their clinical applicability. Many pathological processes, particularly within chronic and slowly expanding lesions, evolve gradually and therefore require extended longitudinal observation to be fully characterized. The weak correlations with EDSS further emphasize the need for more sensitive functional measures, standardized imaging protocols, and the stratification of patient cohorts according to their disease course. By integrating findings across NAWM, GM, and lesional tissue, and by incorporating evidence on clinical correlations and treatment effects, this review offers a more comprehensive perspective than previous work and outlines clear priorities for advancing imaging myelin biomarkers toward clinical translation. Addressing current knowledge gaps will enable MRI to evolve from a primarily observational tool into a predictive tool for disease trajectory, enabling more targeted and personalized therapeutic strategies in MS.

## Data Availability

No data was used for the research described in the article.
